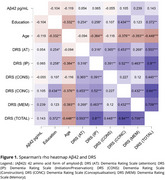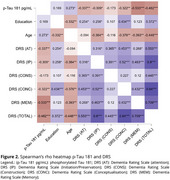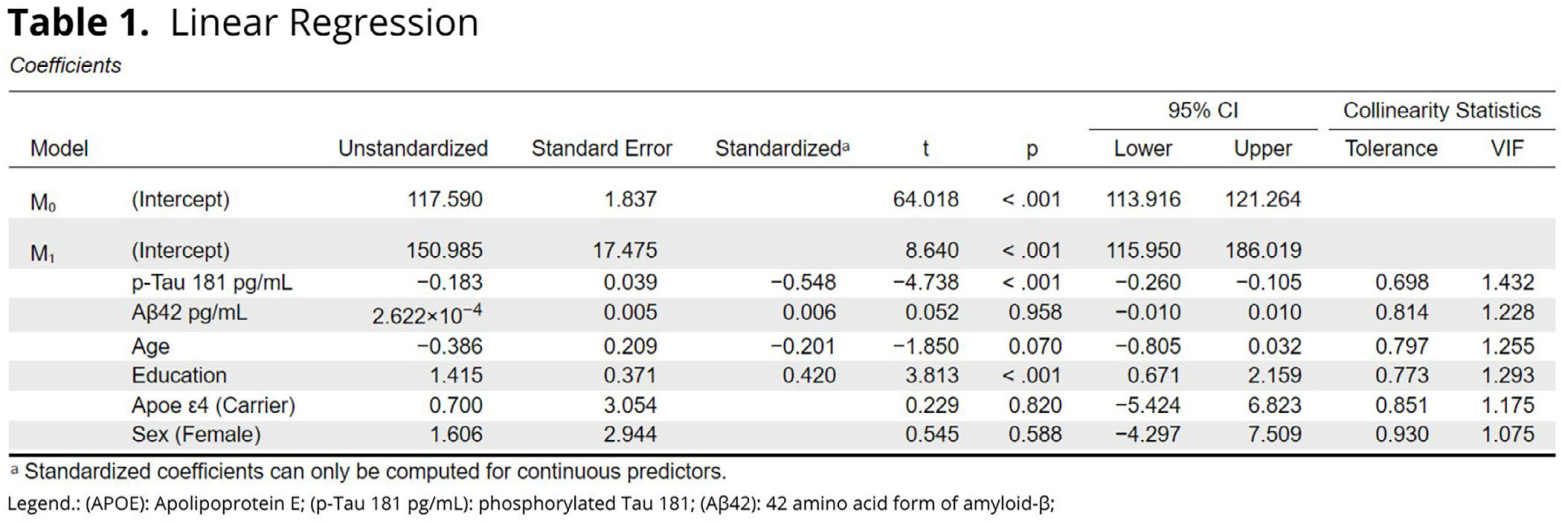# AD biomarkers and DRS performance: Cohort Cog‐Aging, Brazil results

**DOI:** 10.1002/alz70857_106379

**Published:** 2025-12-25

**Authors:** Gabriela Tomé Oliveira Engelmann, Giovanna Correia Pereira Moro, Ivonne Carolina Bolaños Burgos, Júlia de Almeida Barreto, Joice Coutinho de Alvarenga, João Henrique Fonseca, Thaise Vallesca Queiroz, Rafaela Teixeira de Ávila, Erika de Oliveira Hansen, Natália Silva Dias, Marco Aurelio Romano‐Silva, Luiz Armando Cunha de Marco, Bernardo de Mattos Viana, Maria Aparecida Camargos Bicalho

**Affiliations:** ^1^ Older Adult's Psychiatry and Psychology Extension Program (PROEPSI), School of Medicine, Universidade Federal de Minas Gerais (UFMG), Belo Horizonte, Minas Gerais, Brazil; ^2^ Cog‐Aging Research Group, Universidade Federal de Minas Gerais (UFMG), Belo Horizonte, Minas Gerais, Brazil; ^3^ Molecular Medicine Postgraduate Program, School of Medicine, Universidade Federal de Minas Gerais (UFMG), Belo Horizonte, Minas Gerais, Brazil; ^4^ Undergraduate Medicine, Faculty of Medicine, Universidade Federal de Minas Gerais (UFMG), Belo Horizonte, Minas Gerais, Brazil; ^5^ Cog‐Aging Research Group, Belo Horizonte, Minas Gerais, Brazil; ^6^ Neurotec R National Institute of Science and Technology (INCT‐Neurotec R), Faculty of Medicine, Universidade Federal de Minas Gerais (UFMG), Belo Horizonte, Minas Gerais, Brazil; ^7^ Federal University of Minas Gerais, Belo Horizonte, Minas Gerais, Brazil; ^8^ Cog‐Aging Group Research, Brazil, Belo Horizonte, Minas Gerais, Brazil; ^9^ Older Adult Psychiatry and Psychology Extension Program (PROEPSI), Brazil, Belo Horizonte, Minas Gerais, Brazil; ^10^ Geriatrics and Gerontology Center Clinical Hospital of Universidade Federal de Minas Gerais, Belo Horizonte, Minas Gerais, Brazil; ^11^ Older Adult Psychiatry and Psychology Extension Program (PROEPSI), Faculty of Medicine, Universidade Federal de Minas Gerais (UFMG), Belo Horizonte, Minas Gerais, Brazil; ^12^ Department of Mental Health, Faculty of Medicine, Universidade Federal de Minas Gerais (UFMG), Belo Horizonte, Minas Gerais, Brazil; ^13^ Universidade Federal de Minas Gerais, Belo Horizonte, Brazil; ^14^ Neurotec R National Institute of Science and Technology (INCT‐Neurotec R), Faculty of Medicine, Federal University of Minas Gerais, Belo Horizonte, Minas Gerais, Brazil; ^15^ Department of Psychiatry, School of Medicine, Federal University of Minas Gerais, Belo Horizonte, Minas Gerais, Brazil; ^16^ Molecular Medicine Program, School of Medicine, Federal University of Minas Gerais, Belo Horizonte, Minas Gerais, Brazil; ^17^ National Institute of Science and Technology Neurotec R (INCT‐MM), Belo Horizonte, Minas Gerais, Brazil; ^18^ Molecular Medicine Postgraduate Program, Faculty of Medicine, Universidade Federal de Minas Gerais (UFMG), Belo Horizonte, Minas Gerais, Brazil; ^19^ National Institute of Science and Technology Neurotec R (INCT‐MM), Faculdade de Medicina, Universidade Federal de Minas Gerais, Belo Horizonte, Brazil; ^20^ Universidade Federal de Minas Gerais, Belo Horizonte, Minas Gerais, Brazil; ^21^ Jenny de Andrade Faria Institute – Outpatient Reference Center for the Elderly, Universidade Federal de Minas Gerais (UFMG), Belo Horizonte, Minas Gerais, Brazil; ^22^ Department of Clinical Medicine, Faculty of Medicine, Universidade Federal de Minas Gerais (UFMG), Belo Horizonte, Minas Gerais, Brazil; ^23^ Hospital das Clínicas da UFMG, University Hospital, Universidade Federal de Minas Gerais (UFMG), Belo Horizonte, Minas Gerais, Brazil; ^24^ Sciences Applied to Adult Health Postgraduate Program, School of Medicine, Universidade Federal de Minas Gerais (UFMG), Belo Horizonte, Minas Gerais, Brazil

## Abstract

**Background:**

The study of the association of biomarkers and neuropsychological assessment can contribute to the understanding of the biological mechanisms associated with the evolution of the neurodegenerative process, as well as to the definition of risk factors for the conversion of patients with cognitive decline into dementia due to Alzheimer's disease (ADD). Our main objective was to determine the relationship between performance on the Dementia Rating Scale (DRS) and the main biomarkers of Alzheimer's Disease.

**Method:**

Sixty‐four older adults from the Cog‐Aging Brazil cohort with low educational levels were recruited and split into three groups: Cognitively Unimpaired (CU, *n* = 18), Mild Cognitive Impairment (MCI, *n* = 21) and Alzheimer's Disease Dementia (ADD, *n* = 25). Levels of CSF Aβ42 and *p*‐Tau 181 were assessed by Luminex xMAP technique. Descriptive statistics were carried out according to normality tests. Spearman correlation tests were explored to analyze associations between CSF levels of Aβ42, *p*‐Tau181, and *p*‐Tau181/Aβ42 ratio and performance in DRS. Significant associations between *p*‐Tau181 and Aβ42 and total DRS score (DRST) were explored in linear regression analysis adjusted for age, sex, education, and APOE ε4 carrier status. This study was approved by UFMG Ethics Committee.

**Result:**

The median age of the participants was 75 years (IQR 9.25). The education level was 4 years (IQR 4.25), DRST mean 117.73 (14.04). A negative significant correlation was found between CSF *p*‐Tau181 and the domains of DRS: Memory (rho ‐0.533) (p < .001) and DRST (rho ‐0.482) (p < .001). No significant correlation was found for Aβ42. A significant correlation between DRS and *p*‐Tau181/Aβ42 ratio was found in Memory (rho ‐0.430) (p < .001) and DRST (rho ‐0.349) (p < .05). The linear regression model was statistically significant (F = 8.81) (p < .001) with R2 of 0.495. The Linear regression analyses showed a significant association with DRS and *p*‐Tau181(β = ‐0.548; CI 95%: [ ‐0.260, ‐0.105]; (p < .001) and education (β = 0.420; CI 95%: [0.671, 2.159]; (p < .001).

**Conclusion:**

The higher levels of *p*‐Tau181 seem to correlate with a worse performance in Memory and total DRS. Considering DRST, *p*‐tau was still associated with worse performance, even when adjusted by sociodemographic factors.